# Homology Modeling and Virtual Screening Studies of Antigen MLAA-42 Protein: Identification of Novel Drug Candidates against Leukemia—An *In Silico* Approach

**DOI:** 10.1155/2020/8196147

**Published:** 2020-03-16

**Authors:** Ihsan A. Shehadi, Huda R. M. Rashdan, Aboubakr H. Abdelmonsef

**Affiliations:** ^1^Chemistry Department, Faculty of Science, University of Sharjah, Sharjah 27272, UAE; ^2^Chemistry of Natural and Microbial Products Department, Pharmaceutical and Drug Industries Research Division, National Research Centre, Dokki 12622, Cairo, Egypt; ^3^Chemistry Department, Faculty of Science, South Valley University, Qena 83523, Egypt

## Abstract

Monocytic leukemia-associated antigen-42 (MLAA-42) is associated with excessive cell division and progression of leukemia. Thus, human MLAA-42 is considered as a promising target for designing of new lead molecules for leukemia treatment. Herein, the 3D model of the target was generated by homology modeling technique. The model was then evaluated using various cheminformatics servers. Moreover, the virtual screening studies were performed to explore the possible binding patterns of ligand molecules to MLAA's active site pocket. Thirteen ligand molecules from the ChemBank^™^ database were identified as they showed good binding affinities, scaffold diversity, and preferential ADME properties which may act as potent drug candidates against leukemia. The study provides the way to identify novel therapeutics with optimal efficacy, targeting MLAA-42.

## 1. Introduction

Cancer is a result of a progressive accumulation of epigenetic changes and genetic aberrations, which lead to uncontrolled accumulation of blood cells [1]. Leukemia is an incurable disease, characterized by the unrestrained proliferation of abnormal white blood cells, readily spread to the entire body through blood stream and lymphatic system [2]. It starts in blood-forming organs and directs the formation of abnormal blood cells which can divide to produce copies of themselves [3]. Acute monocytic leukemia (AMoL) is an irremediable disease in older adults [4]. The present need is to identify novel and more effective therapies for AMoL to alleviate the suffering of patients.

**Figure 1:**
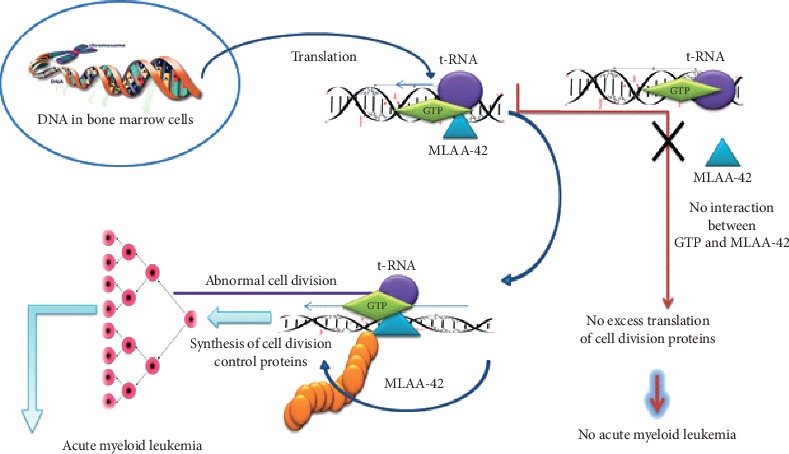
Biochemical pathway of MLAA-42 protein involves transformation of normal cells to malignant phenotype through binding with GTP bound protein leading to unrestrained proliferation of the abnormal white blood cells which lead to AMoL.

MLAA-42 is one of immunogenic antigens and a novel-identified monocytic leukemia-associated antigen which is overexpressed in acute monocytic leukemia. In an attempt to uncover the mechanism of MLAA-42 overexpression, it significantly works as an elongation factor which plays an important role in polypeptide elongation in leukemia as shown in the biochemical pathway (Figure 1) [5]. Furthermore, MLAA-42 plays a critical role in the transformation of normal cells to malignant phenotype through binding with GTP bound protein leading to unrestrained proliferation of the abnormal white blood cells which lead to AMoL. In the meantime, inhibition of proliferation of bone marrow cells, which takes place if there is no binding between MLAA-42 and GTP, is caused directly to prevent formation of AMoL [5].

Heterocyclic compounds have been found to gain extensive interest due to their considerable wide range applications in medicinal, pharmaceutical, and pharmacological activities especially as anticancer agents [6, 7]. In the present study, computer aided virtual screening studies were carried out using ChemBank™ database to report new molecular entities which can be used as potent inhibitors against MLAA-42 protein. This study warrants further investigations to indicate that MLAA-42 is a novel target for designing newer drug-like molecules for leukemia treatment, using in silico approaches.

## 2. Results and Discussion

### 2.1. Generation of the Three-Dimensional Structure of MLAA-42

**Figure 2:**
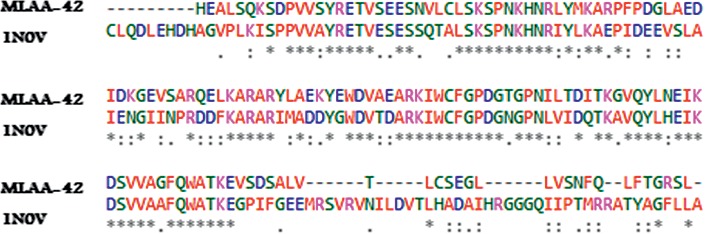
Pairwise sequence alignment of MLAA-42 with 1N0V. The conserved amino acid residues are elucidated as (^*∗*^), highly similar residues as (:), and weakly similar residues as (.).

For the case where the target MLAA-42 is not experimentally determined by means of X-ray crystallography and nuclear magnetic resonance (NMR), the computational-based techniques are used for 3D structure prediction [8]. The FASTA sequence of the target protein (ID : Q6W6M8) with 151 amino acid residues was identified from Universal Protein Resource “UniProtKB” database (https://www.uniprot.org/uniprot/Q6W6M8) [9]. The template was recognized by subjecting the obtained sequence to BLAST, Jpred3, and Domain Fishing servers, and Table 1 represents the resulted maximum *E*-value. The 1N0V “Crystal structure of elongation factor 2” was selected as a template protein, on the basis of parameters such as query cover (77%), maximum identity (63%), and statistical *E*-value (8 × 10^−47^) with the target [10]. ClustalW server was used to perform the alignment of both elongation factor proteins MLAA-42 and 1N0V (541-720 aa), as shown in Figure 2. The conserved amino acid residues are elucidated as (^*∗*^) (39.3%), highly similar residues as (:), and weakly similar residues as (.) (51.7%). The individual percentages, resulted from the server, refer to the identity and similarity of the target residue sequence with its template sequence, respectively.

**Figure 3:**
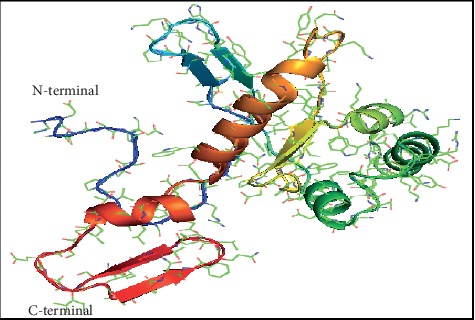
3D structure of MLAA-42 protein. It consists of five *α*-helices and six *β*-strands.

**Figure 4:**
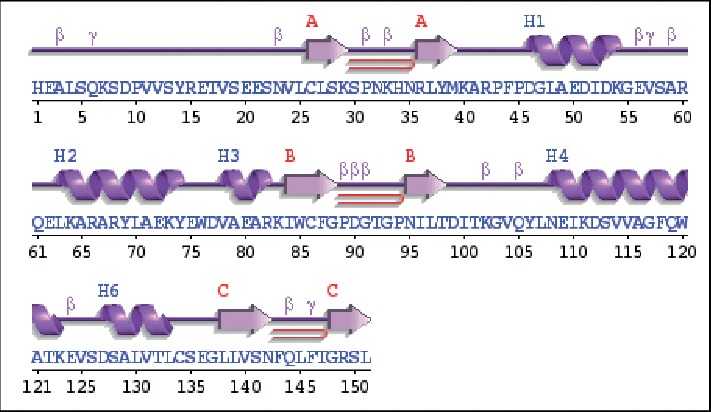
The secondary structure of the target MLAA-42 obtained by the PDBsum server.

**Figure 5:**
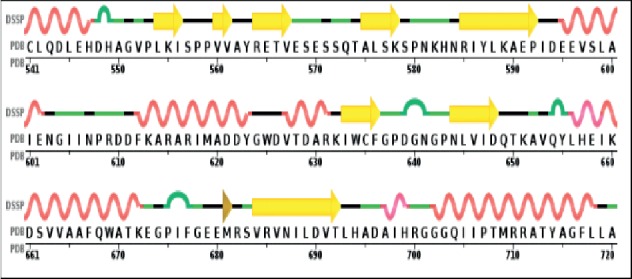
The secondary structure of the template 1N0V (541-720 aa) using the PDBsum server.

**Figure 6:**
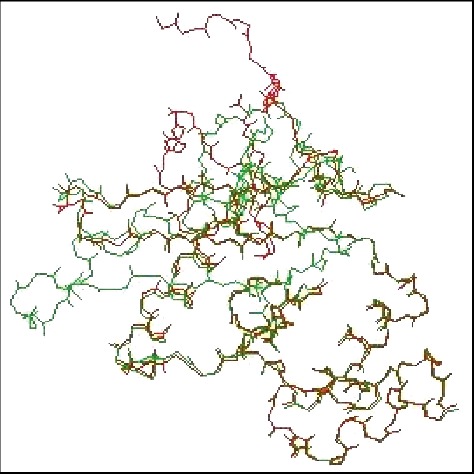
Superimposition of MLAA-42 (red) on template 1N0V (green) in the Swiss- PDB viewer.

Twenty-five models of MLAA-42 were generated by using Modeller 9.11 software, depending on satisfying spatial restraints in terms of probability density function [11]. The 3D model with fewest restraint violations, lowest probability density function (855.33), and acceptable geometry was selected for further validation [12].  Figure 3 represents the 3D model of the target, which consists of five alpha-helices and six beta-strands. The secondary structures of both 1N0V and MLAA-42, which represent the amino acid sequences of helices and strands in both proteins, are represented in Figures 4 and 5 and ([Supplementary-material supplementary-material-1] and Tables [Supplementary-material supplementary-material-1] and [Supplementary-material supplementary-material-1]) in the supplementary data section, using PDBsum server [13]. To obtain a stable confirmation, energy minimization and protein loop modeling were performed by GROMOS96 Force-Field set, using Swiss-Pdb Viewer [14]. To get insight of the superimposition between the proteins MLAA-42 and 1N0V, the root-mean-square deviation RMSD for the backbone of MLAA-42 and 1N0V is 0.51 Å (within the permissible range i.e. ≤2 Å), which indicates close homology and ensures reliability of the model, as declared in Figure 6.

### 2.2. Physicochemical Properties and the Abundance of Amino Acids in MLAA-42

**Figure 7:**
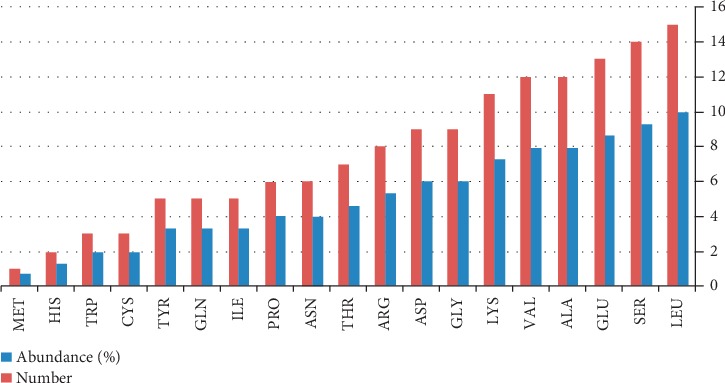
The abundance of amino acids in MLAA-42. Graphical representation of the abundance of 20 amino acids present in the target. Lucine has the highest abundance (9.9%), and methionine has the lowest abundance (0.7%).

The physicochemical properties of MLAA-42 were predicted using ProtParam tool [15]. The protein sequence has 151 amino acid residues with molecular weight of 16.871 kDa. Furthermore, the most abundant amino acid residues are LEU, SER, GLU, ALA, VAL, LYS, GLY, and ASP, respectively, in high percentages in the target protein, as shown in Figure 7. Lucine has the highest abundance (9.9%), and methionine has the lowest abundance (0.7%). The physicochemical parameters predicted a negatively charged protein as the result of the high number of negatively charged residues (aspartic acid 6.0% and glutamic acid 8.6%) in contrast with positively charged ones (arginine 5.3% and lysine 7.3%). The atomic composition of MLAA-42 is 2364, with 747 carbon (C), 1176 hydrogen (H), 204 nitrogen (N), 233 oxygen (O), and 4 sulfur (S). In addition, the protein is acidic, with an isoelectric point (pI) of 5.47. The estimated half-life of the target showed that it can remain intact without being degraded for 3.5 h in humans, 10 min in yeast, and less than 10 h in *E. coli*, and its extinction coefficient is 24075 M^−1 ^cm^−1^. Finally, the generated aliphatic index was 82.65, with grand average of hydrophobicity (GRAVY) of −0.426 and an instability index of 34.55.

### 2.3. Validation of the Model and Active Site Analysis

**Figure 8:**
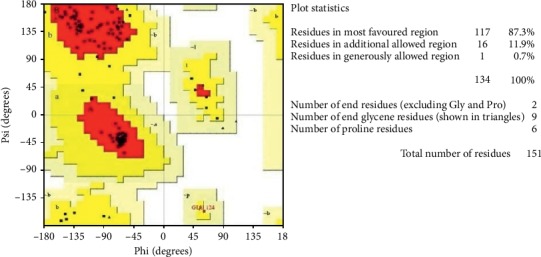
Stereochemical analysis of MLAAA-42. The red region declares the most favorable area of residues; the yellow region is additionally allowed; and generously allowed residues in the light-yellow region. The RC plot declares 99.2% of residues falling in the allowed region.

The 3D model was validated by different tools such as PROCHECK (Ramachandran plot) and ProSA to check its structural integrity. Ramachandran plot (Figure 8) of the modeled protein represents 87.3% (117 aa) of the total residues in the most favored regions and 11.9% (16 aa) in additionally allowed regions, indicating a good quality model.

Moreover, ProSA Z-score (dark spot) is −2.85, which falls within the values range of the known proteins determined by X-ray (light blue) and NMR (dark blue) (see [Supplementary-material supplementary-material-1], in the supplementary data section), predicting a reasonable quality model. [Supplementary-material supplementary-material-1], in the supplementary data section, shows the 3D model quality by plotting energies (*X*-axis) as a function against residue sequence position (*Y*-axis). The figure shows that most of residues of MLAA-42 have negative energies, which indicates an acceptable model. In view of the facts mentioned above about the unavailability of 3D structure of MLAA-42 in protein data bank PDB, thus, there are no previous data obtained for the binding regions of the target. Therefore, to overcome the lack of experimental data, herein, CASTp and SiteMap cheminformatics tools were used to identify the binding site cavities of the target (Figures [Supplementary-material supplementary-material-1] and [Supplementary-material supplementary-material-1] and Tables [Supplementary-material supplementary-material-1] and [Supplementary-material supplementary-material-1]), in the supplementary data section, respectively. CASTp server gives information about hydrophobic pocket regions; in addition, it measures cavity volume and area. The results declared four sites, site 1 has volume of 40.9 Å^3^, site 2 with volume of 28.2 Å^3^, site 3 with volume of 50.7 Å^3^, and site 4 has volume of 105.4 Å^3^. Among the four different cavities obtained from CASTp, the amino acids from PHE44 to ILE96 are consistently present in all sites. SiteMap gives information about the hydrophilic and hydrophobic regions present on the protein surface. It identified two binding cavities on MLAA-42 protein. The site 1 with volume 1222.79 Å^3^ and size of 4.38 Å and site 2 has volume of 46.30 Å^3^ and size of 32 Å. The active sites predicted by SiteMap are from TYR38 to ILE100. In conclusion, these computational prediction techniques give information about the binding site regions that are composed of TYR38 to ILE100 residues are responsible for MLAA-42 docking with ligand molecules. The docking interactions show that the residues TYR38, LYS40, THR89, ILE96, ASP99, and ILE100 are observed to be crucial to identify the antagonists against MLAA-42 protein. [Supplementary-material supplementary-material-1], in the supplementary data section, represents the grid with dimensions 80 Å × 80 Å × 80 Å around the binding site, to perform further studies.

### 2.4. Molecular Docking Analysis and ADMET Profile

The compounds with heterocyclic core structures are pharmaceutically important class of compounds because of their diverse range of biological importance such as anticancer activity against cancer cell lines [6, 7]. In view of the facts mentioned above and as a part of our efforts to identify new anticancer drug candidates [16], a ChemBank™ library of 2344 small molecules with an anticancer activity was downloaded [17] for specific docking. Computer-aided screening protocol at the binding regions of the target was performed using virtual screening workflow docking program of Glide, Schrodinger suite. The glide docking program identifies specific structural motifs and provides exceptionally large contribution to enhance binding affinity [18]. The docking approach was run in a flexible docking mode which automatically generates different confirmations for each ligand molecule. The total isomeric structures (13,354) obtained after ligand preparation was subjected to high throughput virtual screening (HTVS) mode and 10% hit structures (1335) obtained as output, followed by 10% of them, 133, screened in standard precision (SP) mode, and finally the 10% of the resultant were screened in the Extra precision (XP) mode to obtain 13 hits. The docked compounds were prioritized according to their binding energies (in range of −12.19 to −9.22 Kcal/mol) after docking to the active site pocket of the target protein, as tabulated in Table 2.

**Figure 9:**
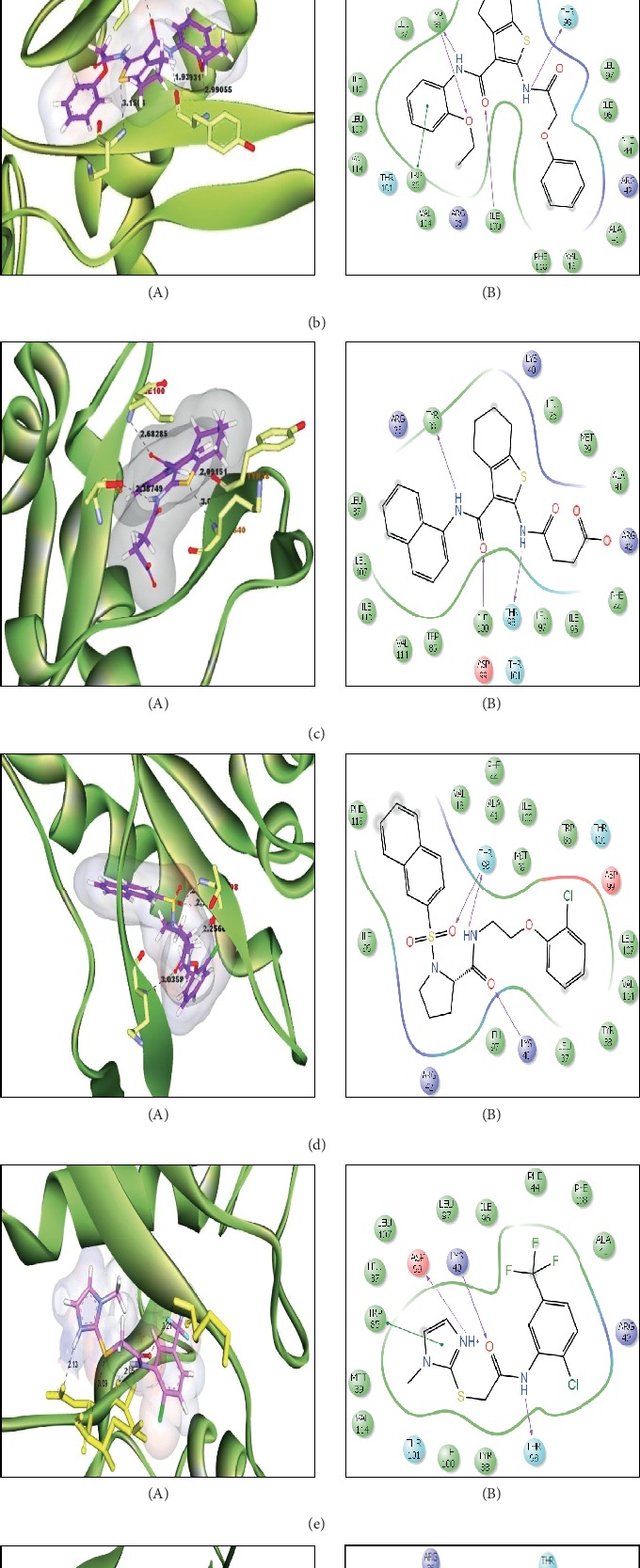
Interactions between MLAA-42 with ligand molecules (a)–(f). (A) Three-dimensional docked poses: the binding residues of MAA-42 are represented in yellow stick, the ligand molecules are shown in the purple stick model, the hydrogen bonds are shown in black dotted lines, and pi-pi interactions in orange lines. (B) Two-dimensional docked poses: the residues are shown in a 3-letter code, hydrogen bonds in pink lines, and pi-pi interactions in green lines.

A sample of six best docking interactions between the ligands and protein, arranged rankwise based on their binding energies, is depicted in Figure 9 and Table 2. The top six ligand-protein complexes were visually checked in Discovery Studio Visualizer (for 3D representation) and Maestro (for 2D representation), which exhibited the noncovalent interactions such as hydrogen bonding and pi-pi stacking [19, 20]. In 3D representation, the binding residues of MLAA-42 are shown in yellow-colored stick model, the ligands in purple one, H-bonds are in black dotted lines, and pi-pi interactions are in orange lines (Figure 9). While in 2D representation, the residues are shown in a 3-letter code, the hydrogen bonds are represented in pink lines, and pi-interactions are shown in green lines. The ligand molecules with heterocyclic moieties such as thiophene, pyrazole, and pyrolidine and amide groups such as carboxamide (-CONH-) and sulphonamide (-SO_2_NH-) in ligands L1-L6 are observed to be common pharmacophore groups which interact with the active site region (TYR38, LYS40, TRP 85, ILE96, THR98, ASP99, and ILE100) of MLAA-42 through a network of hydrogen bonds and pi-interactions, as represented in Table 2. The carboxamide group, which is present in all ligand molecules, formed intermolecular hydrogen bonds with the residues TYR38, LYS40, TRP 85, ILE96, THR98, ASP99, and ILE100. In addition, the sulphonamide group, which is present in ligand L4, formed a hydrogen bond interaction (N---H---O) with the residue THR98 at the distance of 2.65 Å. Furthermore, the tryptophan (TRP85) formed *π*-*π* interactions (face to face) with the benzene ring of ligand L2 on its side chain plus the pyrazole ring of ligand L5, at the distance of 4.95 and 4.87  Å, respectively. The intermolecular hydrogen bonds and pi-interactions add more stability to the docked complexes [21]. The other docked compounds to the target are included in supplementary data section as [Supplementary-material supplementary-material-1].

SASA values of MLAA-42, before and after docking, were analysed using Discovery Studio Visualizer (see [Supplementary-material supplementary-material-1], in the supplementary data section). The decrease in SASA values confirms that the amino acid residues (TYR38 to ILE100) are involved in bonds formation with ligand molecules (a)–(f).

The ADMET properties for the resulted ligand molecules were determined in silico by using the QikProp module of the Schrödinger suite. The molecules with agreeable ADME properties are considered as new novel drug candidates, as shown in Table 3. Interestingly, all the compounds have a molecular weight in the acceptable range of 349.7 to 511.3 Daltons (less than 725 Dalton). BBB^+^ value describes the ability of the compounds to cross the blood-brain barrier, which is in the permissible ranges for all compounds. They had <10 hydrogen bond acceptors and <5 hydrogen bond donors, and log *P* values of <5. These properties are in the reasonable range of Lipinski's rule of five (LORF) [22]. The human oral absorption, partition coefficient (*Q*Plog*P*o/w), and water solubility (*Q*PlogS) values are in the agreeable range defined for human use, which indicates their possible ability to be drug candidates, as shown in Table 3. Also, the values show that the compounds can be absorbed by the human intestines. Furthermore, all molecules (a)–(f) displayed negative AMES toxicity test which means that the ligands are nonmutagenic. Also, they displayed negative carcinogenicity test. These ligands can be considered as highly potent inhibitors against leukemia.

## 3. Materials and Methods

### 3.1. 3D Homology Model of MLAA-42

The protein's three-dimensional structure is required to understand its function [23]. Herein, an in silico homology modeling generates a three-dimensional (3D) for an unknown structure of protein (target) depending on one or more proteins of known structures (templates) as reported earlier by Aboubakr et al. in 2016 [24, 25]. The generation of (3D) structure of MLAA-42 was carried out using comparative modeling techniques. The amino acid sequence of MLAA-42 is retrieved from the UniProtKB database in FASTA format. The resulted sequence is submitted to BLASTp, Jpred3, and Domain fishing servers, to identify suitable templates [26–28]. E-value is identified the similarity between the target and template proteins [29]. The template with maximum identity is selected for generating the 3D structure of the target. The pairwise sequence alignment of MLAA-42 with its template is performed using the ClustalW tool to recognize the structurally conserved regions [30]. ClustalW server uses the Gonnet matrix algorithm to make certain the presence of conserved motifs and to minimize the atomic gaps. The % identity of alignment is measured as a ratio number of the identical residues in the alignment and the total number of residues of the target. The % similarity is measured as a ratio of the total number of identical and similar residues to the total number of residues of the target. The 3D structure of the target is generated using Modeler 9.11. Then, the model is energy minimized using SPDBV and Impref in Schrödinger to obtain a stable conformation [31]. The RMSD value, which is calculated using the Swiss-Pdb viewer, is used to find the best superimposition for protein structures. The lower value (≤2 Å) means the best alignment between the structures.

### 3.2. 3D Structure Validation

Subsequently, quality of the generated model is evaluated by computational protocols such as PROCHECK and Protein Structural Analysis (ProSA) [32, 33]. PROCHECK, a program that relies on Ramachandran plots for structure verification, figures out the stereochemical quality of the model. Furthermore, ProSA is applied to check for energy criteria in comparison with a large set of known protein structures with similar size [34].

### 3.3. Binding Site Prediction and Ligand Preparation

It is the hydrophobic cavities on the surface of a protein, which is responsible for its specificity [35]. It is theoretically determined by means of tools based on recent theoretical and algorithmic results of computational geometry such as CAST-p and SiteMap of Schrödinger suite [36, 37]. These computational prediction tools analytically furnished the area and volume of cavities. The grid is created around the binding cavity to perform further structure-based virtual screening studies [38]. *LigPrep* module of Schrödinger suite is used to generate various conformers of small molecule depending upon its structural features [39].

### 3.4. In silico Molecular Docking, SASA, and ADME Analysis

Molecular docking protocol is used to predict the preferred orientation of a ligand molecule to the target protein to form a stable complex [40, 41]. The docking accuracy is determined by finding how closely the binding confirmation with the lowest energy of the cocrystalized ligand molecule predicted by the object scoring function; G-score (Glide score) resembles an experimental binding modes determined by X-ray crystallographic technique [42]. Once the 3D model of MLAA-42 is validated and the target binding pockets are defined, the docking-based virtual screening study is performed using the Glide docking tool incorporated in the Schrödinger package by Maestro [43]. The virtual screening approach is performed through hierarchal flexible docking methods: High Throughput Virtual Screening (HTVS), Standard Precision (SP), and Extra Precision (XP). The ligand molecules are prioritized on the basis of the docking score, docking energy in each step, and by default 10% of the molecules selected and considered for the next hierarchical step [18]. Solvent Accessible Surface Area (SASA) of the target, before and after docking, is computed using Accelrys Discovery Studio Visualizer 3.5 software. Prediction of drug-like profiles, such as physicochemical, pharmacokinetic, and safety of the compounds, is performed using the QikProp module [44].

## 4. Conclusion

In this work, the computer-aided drug design protocols were used to identify novel leads against MLAA-42 protein. The homology model of the target was evaluated by homology modeling techniques. In silico molecular docking were also adopted to identify the lead compounds. The resulted molecules with heteroscaffolds and amide groups (-CONH-and–SO_2_NH-) exhibited better estimated binding energy values and agreeable pharmacokinetic properties and were ranked as potent drug-like candidates against the target protein. Hence, MLAA-42 has emerged as a therapeutic target for treatment of leukemia carcinoma.

## Figures and Tables

**Table 1:** The template protein selection.

Server name	Parameters for template selection	*E*-values	PDB code
NCBI-Blast	Sequence similarity	9e^−47^	1N0V
Jpred3	Secondary structure prediction	6e^−40^	1N0V
Domain fishing	Domain similarity	3e^−40^	1N0V

1N0V was selected as a template protein using different servers, based on query cover, maximum identity, and statistical *E*-value.

**Table 2:** The chemical structures of top six scoring ligand molecules against MLAA-42 protein.

S. no.	Structure and IUPAC name of molecule	Glide energy (KJ/Mol)	Binding energy (Kcal/mol)	The interacting amino acid residues	Bond distance (Å)
1	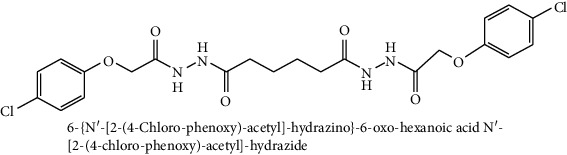	−63.59	−12.19	H-bonds	
				LYS40 : N – L1	2.91
				THR98 : N – L1	2.50
				ILE100 : N – L1	2.67
				LYS40 : O – L1	2.32
				ILE96 : O – L1	2.18
				THR98 : O – L1	2.15

2	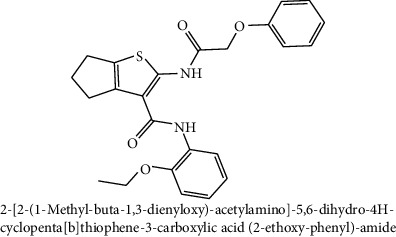	−50.27	−11.83	H-bonds	
				TYR38 : N – L2	2.89
				ILE100 : N – L2	2.99
				TYR38 : O – L2	1.94
				*π*-*π* interactions	
				TRP85-L2	4.95

3	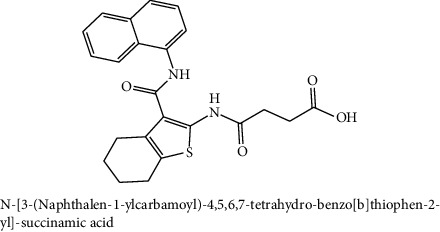	−42.22	−11.61	H-bonds	
				ILE100 : H – L3	1.68
				THR98 : O – L3	2.36
				TYR38 : O – L3	2.00

4	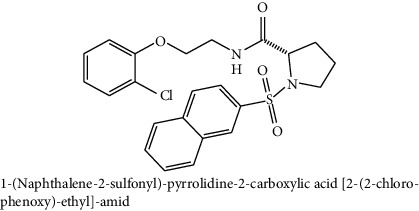	−47.52	−11.30	H-bonds	
				LYS40 : N – L4	2.95
				THR98 : N – L4	2.65
				THR98 : O – L4	2.26

5	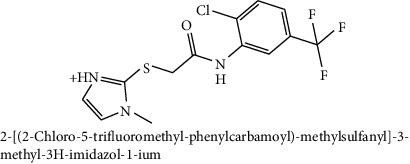	−39.53	−11.12	H-bonds	
				LYS40 : N – L5	2.67
				THR98 : O – L5	2.13
				ASP99 : OD1-L5	2.79
				π-π interactions	
				TRP85-L5	4.87

6	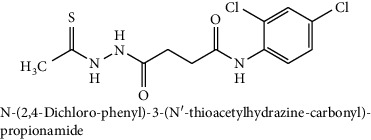	−40.65	−11.10	H-bonds	
				ILE100 : N – L6	2.69
				TYR38 : O – L6	2.30
				THR98 : OG1-L6	2.29

Table represents top six ligand molecules with binding energies in range of −12.19 to −9.22 Kcal/mol, hydrogen bonds, and pi-interactions to MLAA-42's active site pocket.

**Table 3:** ADME properties of the top docked ligand molecules.

Molecule	CNS	M. (Wt)	SASA	Volume	Donor HB	Accept HB	QPlogPo (w)	QPPCaco	QPlogBB	Percent human oral absorption	^#^NandO	Rule of five	Rule of three
L1	−2	511.3	905.7	1554.4	1	8	4.7	64.5	−2.8	74.0	10	1	1
L2	0	436.5	751.0	1348.2	1	5	5.5	2264.9	−0.5	100	6	1	2
L3	−2	422.4	740.5	1298.5	2	6	4.4	48.1	−1.7	83.1	6	0	1
L4	−1	458.9	755.6	1350.7	1	8	3.9	883.3	−0.6	100	6	0	0
L5	1	349.7	564.0	962.4	1	4	4.1	2206.4	−0.2	100	4	0	0
L6	−1	411.3	708.3	1210.2	3	7	3.9	538.7	−0.8	100	6	0	1

The agreeable ranges are as follows: CNS: −2 (inactive), +2 (active). Mol wt: (130–725). Volume: (500-2000). Donor HB: (0.0–6.0). Accept HB: (2.0–20.0). QPlogPo/w: (-2.0 to 6.5). QPPCaco: <25 poor, >500 great. QPlogBB: (−3.0 to −1.2). % human oral absorption: >80% high, <25% low. Rule of three (3). Rule of five (4).

## Data Availability

The data used to support the findings of this study are included within the article.

## Abbreviations

MLAA-42: Monocytic leukemia-associated antigen-42
UniProt 1N0V: Universe protein resource crystal structure of elongation factor 2
3D: Three-dimensional
GEF: Guanine nucleotide exchange factors
GAP: GTPase-activating proteins
BLASTp: Basic alignment tool program
Jpred3: Java prediction V 3.0
Impef: Impact refinement module
ProSA: Protein structure analysis
Castp: Computed atlas of surface topography of protein
RMSD: Root-mean-square deviation
SPDV: Swiss PDB viewer
OPLS: Optimized potentials for liquid simulations
PPIs: Protein-protein interactions
SASA: Solvent accessible surface area
ADME: Adsorption, distribution, metabolic, and excretion.

## Supplementary Materials

Supplementary Materials Figure S1: the secondary structure of MLAA-42. The 3D model of the target protein consists of five helices as red cylinders and six strands as pink arrows. Figure S2: The 3D model quality of MLAA-42. The score (−2.85) is falling in the range of PDB proteins identified by NMR (dark-blue region) and X-ray crystallography (light-blue region). Figure S3: ProSA of MLAA-42 protein. The plot shows local model quality by plotting energies as a function against amino acid sequence position. Figure S4: binding site of MLAA-42 from the CAST-p server. Figure S5: binding site of MLAA-42 by SiteMap module. Figure S6: receptor grid generation by Schrodinger Suite. The grid generated with 80 Å × 80 Å × 80 Å dimensions using receptor grid generation of Glide module. Figure S7: two-dimensional interactions between MLAA-42 with other ligand molecules. Figure S8: the surface accessibility of MLAA-42 and docked complex (L1). Red-colored peaks represent SASA value of MLAA-42 (after docking), blue-colored peaks represent before docking. *X*-axis represents the amino acid residues and *Y*-axis represents SASA value. Tables S1 and S2: the secondary structure details of MLAA-42 protein (S1) the *α*-helices in MLAA-42 protein. (S2) the *β*-strands in MLAA-42 protein. Table S3: binding site of the MLAA-42 by CAST-p server. Table S4: binding site of MLAA-42 by SiteMap module.
