# Supplementary material for: Homology Modeling and Virtual Screening Studies of Antigen MLAA-42 Protein: Identification of Novel Drug Candidates against Leukemia—An In Silico Approach
Source: Comput Math Methods Med. 2020 Mar 16;2020:8196147. doi: 10.1155/2020/8196147 (PMC7102452; doi:10.1155/2020/8196147)
Supplement: Supplementary Materials — Figure S1: the secondary structure of MLAA-42. The 3D model of the target protein consists of five helices as red cylinders and six strands as pink arrows. Figure S2: The 3D model quality of MLAA-42. The score (−2.85) is falling in the range of PDB proteins identified by NMR (dark-blue region) and X-ray crystallography (light-blue region). Figure S3: ProSA of MLAA-42 protein. The plot shows local model quality by plotting energies as a function against amino acid sequence position. Figure S4: binding site of MLAA-42 from the CAST-p server. Figure S5: binding site of MLAA-42 by SiteMap module. Figure S6: receptor grid generation by Schrodinger Suite. The grid generated with 80 Å × 80 Å × 80 Å dimensions using receptor grid generation of Glide module. Figure S7: two-dimensional interactions between MLAA-42 with other ligand molecules. Figure S8: the surface accessibility of MLAA-42 and docked complex (L1). Red-colored peaks represent SASA value of MLAA-42 (after docking), blue-colored peaks represent before docking. X-axis represents the amino acid residues and Y-axis represents SASA value. Tables S1 and S2: the secondary structure details of MLAA-42 protein (S1) the α-helices in MLAA-42 protein. (S2) the β-strands in MLAA-42 protein. Table S3: binding site of the MLAA-42 by CAST-p server. Table S4: binding site of MLAA-42 by SiteMap module. [file 8196147.f1.docx]

**Homology Modeling and Virtual Screening Studies of Antigen MLAA-42 protein-**

**Identification of Novel Drug Candidates against Leukemia: An *In silico* Approach**

Ihsan Shehadi ^1^, Huda R.M. Rashdan ^2^, Aboubakr H. Abdelmonsef ^3*^

^1^ Chemistry Department, Faculty of Science, University of Sharjah, Sharjah 27272, UAE; [ishehadi@sharjah.ac.ae](mailto:ishehadi@sharjah.ac.ae)

^2^ Chemistry of Natural and Microbial Products Department, Pharmaceutical and Drug Industries Research Division, National Research Centre, 12622 Dokki, Cairo, Egypt; [hudadawoud20@yahoo.com](mailto:hudadawoud20@yahoo.com)

^3^ Chemistry Department, Faculty of Science, South Valley University, 83523 Qena, Egypt;

[aboubakr.ahmed@sci.svu.edu.eg](mailto:aboubakr.ahmed@sci.svu.edu.eg)

* Correspondence: e-mail: [aboubakr.ahmed@sci.svu.edu.eg](mailto:aboubakr.ahmed@sci.svu.edu.eg); Tel: (+2 01098965494).

**Supplementary information**


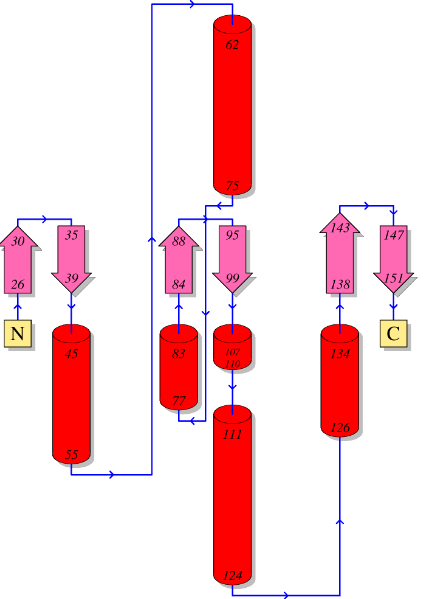


**Figure S1.**The secondary structure of MLAA-42. The 3D model of the target protein consists of five helices as red cylinders and six strands as pink arrows.

**
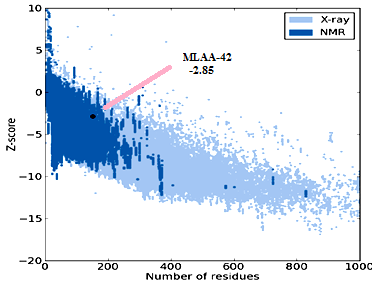
**

**Figure S2.** The 3D model quality of MLAA-42. The score (-2.85) is falling in the range of PDB proteins identified by NMR (dark blue region) and X-Ray crystallography (light blue region).

**
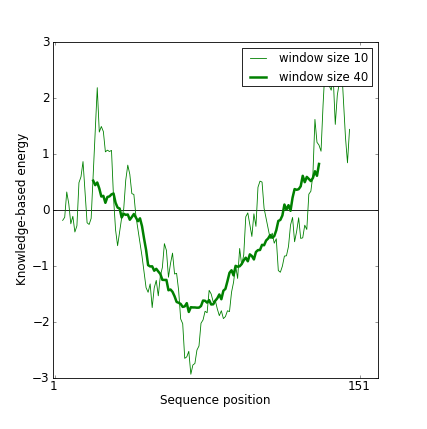
**

**Figure S3.** ProSA of MLAA-42 protein. The plot shows local model quality by plotting energies as a function against amino acid sequence position.


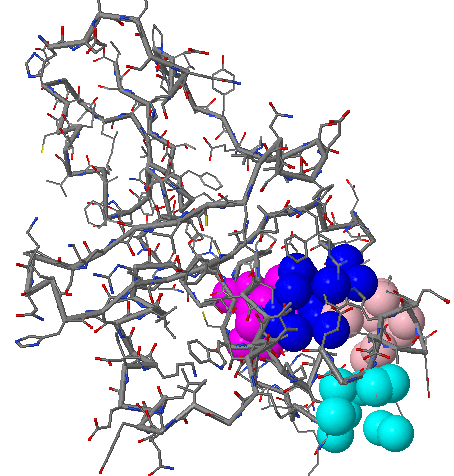


**Figure S4.** Binding site of MLAA-42 from the CAST-p server


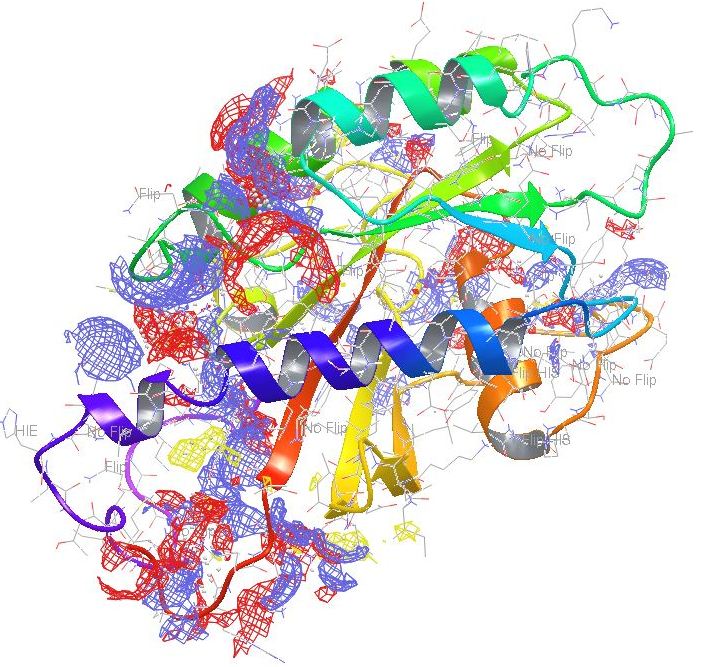


**Figure S5.** Binding site of MLAA-42 by SiteMap module

**
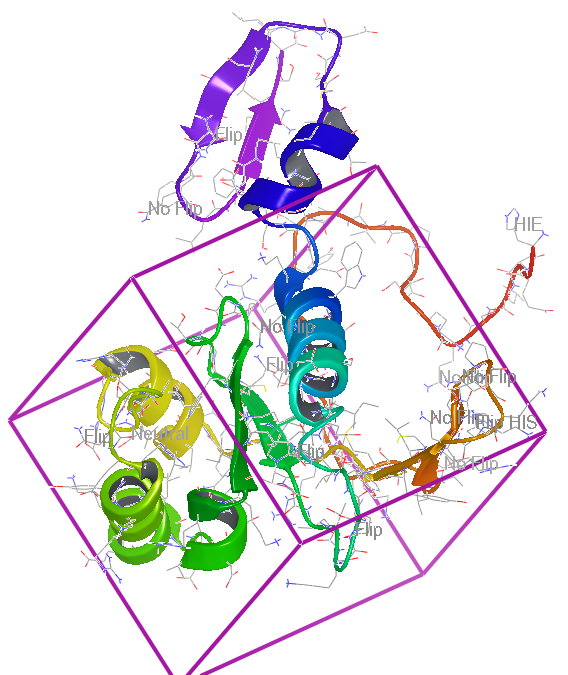
**

**Figure S6.** Receptor grid generation by Schrodinger Suite. The grid generated with 80Å ×80Å ×80Å dimensions using receptor grid generation of Glide module.

| **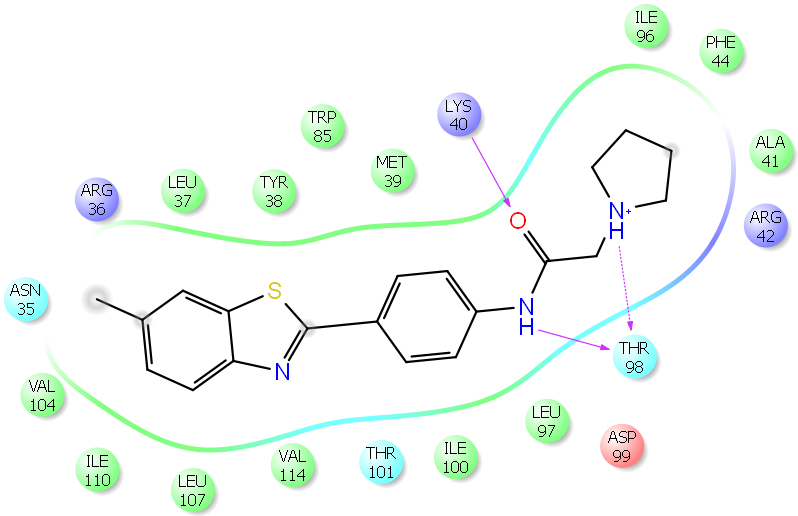**  **L7** |
| --- |
| **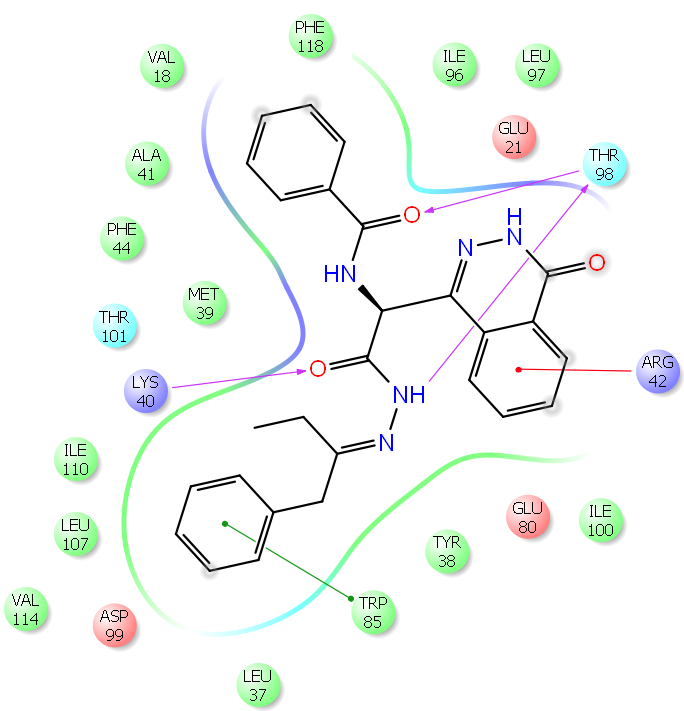**  **L88** |
| **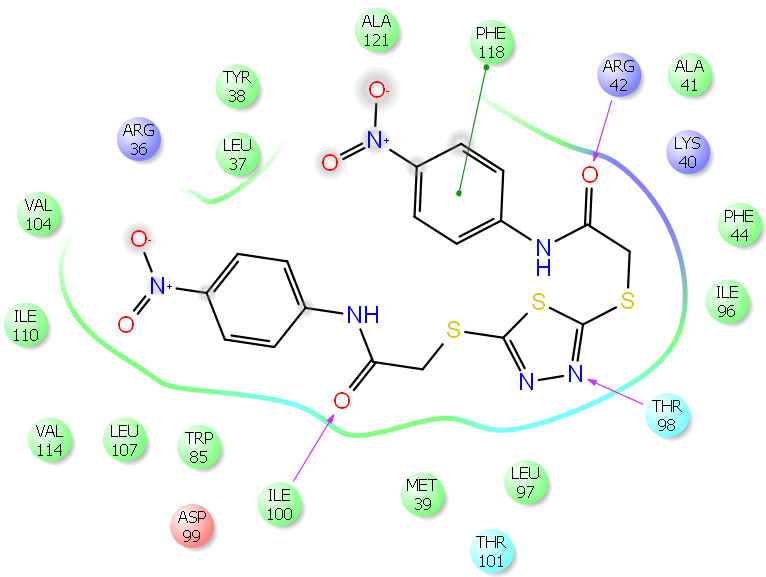**  **L9** |

**Figure S7.** Two-dimensional interactions between MLAA-42 with other ligand molecules.

**Figure S8.**The surface accessibility of MLAA-42 and docked complex (L1). Red colored peaks represent SASA value of MLAA-42 (after docking), blue colored peaks represent before docking. X-axis represents the amino acid residues and Y-axis represents SASA value.

**Tables S1 and S2.** The secondary structure details of MLAA-42 protein

**(S1) The α-helices in MLAA-42 protein**

| **S.No.** | **Start amino acid** | **End**  **amino acid** | **No. of residues** | **Length of**  **helix (A°)** | **Amino acid sequence** |
| --- | --- | --- | --- | --- | --- |
| 1 | Asp46 | Lys54 | 9 | 11.58 | DGLAEDIDL |
| 2 | Leu63 | Lys73 | 11 | 15.05 | LKARARYLAEK |
| 3 | Val78 | Lys83 | 6 | 8.4 | VAEARK |
| 4 | Asn108 | Lys123 | 16 | 23.28 | NEIKDSVVAGFQWATK |
| 5 | Ser128 | Thr132 | 5 | 8.28 | SALVT |

**(S2) The β-strands in MLAA-42 protein**

| **S.No.** | **Start**  **amino acid** | **End**  **amino acid** | **No. of residues** | **Length of strand (A°)** | **Amino acid sequence** |
| --- | --- | --- | --- | --- | --- |
| 1 | Cys26 | Ser30 | 5 | 13.40 | CLSKS |
| 2 | Asn35 | Met39 | 5 | 13.36 | NRLYM |
| 3 | Lys83 | Asp90 | 8 | 18.73 | KIWCFGPD |
| 4 | Thr92 | Asp99 | 8 | 17.50 | TGPNILTD |
| 5 | Ser141 | Phe143 | 3 | 6.61 | SNF |
| 6 | Thr147 | Arg149 | 3 | 6.86 | TGR |

**Table S3.** Binding site of the MLAA-42 by CAST-p server

| **Site No.** | **Volume (A°)^3^** | **Residues** |
| --- | --- | --- |
| Site 1 | 40.9 | 44, 48, 52, 76, 96 |
| Site 2 | 28.2 | 52, 57, 59, 87, 92 |
| Site 3 | 50.7 | 64, 67, 68, 78, 82 |
| Site 4 | 105.4 | 48, 52, 57, 70, 76, 84, 87 |

CAST-p server represents four binding sites, volumes and the amino acid residues.

**Table S4.** Binding site of MLAA-42 by SiteMap module

| **Site** | **Site score** | **Size** | **Volume (A°^3^)** |
| --- | --- | --- | --- |
| SiteMap_site_1 | 1.00 | 483 | 1222.79 |
| SiteMap_site_2 | 0.63 | 32 | 46.30 |

SiteMap server represents two binding sites, volumes and sizes.
